# Experiences, impacts and mental health functioning during a COVID-19 outbreak and lockdown: Data from a diverse New York City sample of college students

**DOI:** 10.1371/journal.pone.0249768

**Published:** 2021-04-07

**Authors:** Teresa López-Castro, Laura Brandt, Nishanthi J. Anthonipillai, Adriana Espinosa, Robert Melara

**Affiliations:** 1 Psychology Department, The City College of New York, New York, New York, United States of America; 2 Division on Substance Use Disorders, New York State Psychiatric Institute and Department of Psychiatry, Columbia University Medical Center, New York, New York, United States of America; Unviersity of Sheffield, UNITED KINGDOM

## Abstract

In March 2020, New York City (NYC) experienced an outbreak of coronavirus disease 2019 (COVID-19) which resulted in a 78-day mass confinement of all residents other than essential workers. The aims of the current study were to (1) document the breadth of COVID-19 experiences and their impacts on college students of a minority-serving academic institution in NYC; (2) explore associations between patterns of COVID-19 experiences and psychosocial functioning during the prolonged lockdown, and (3) explore sex and racial/ethnic differences in COVID-19-related experiences and mental health correlates. A total of 909 ethnically and racially diverse students completed an online survey in May 2020. Findings highlight significant impediments to multiple areas of students’ daily life during this period (i.e., home life, work life, social environment, and emotional and physical health) and a vast majority reported heightened symptoms of depression and generalized anxiety. These life disruptions were significantly related to poorer mental health. Moreover, those who reported the loss of a close friend or loved one from COVID-19 (17%) experienced significantly more psychological distress than counterparts with other types of infection-related histories. Nonetheless, the majority (96%) reported at least one positive experience since the pandemic began. Our findings add to a growing understanding of COVID-19 impacts on psychological health and contribute the important perspective of the North American epicenter of the pandemic during the time frame of this investigation. We discuss how the results may inform best practices to support students’ well-being and serve as a benchmark for future studies of US student populations facing COVID-19 and its aftermath.

## Introduction

**Fig 1:**
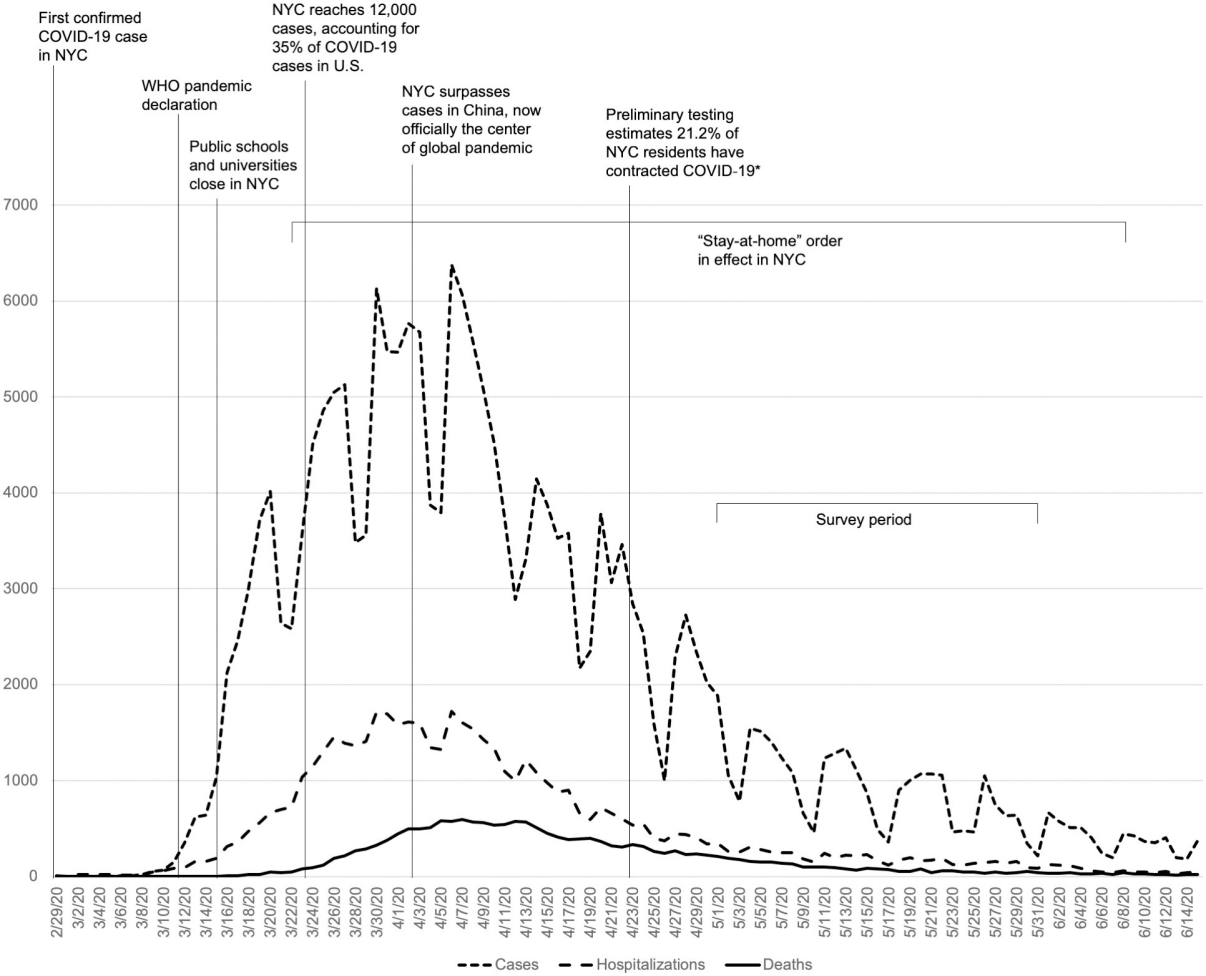
Timeline of COVID-19 events related to New York City (NYC) between February 29 and June 15, 2020, superimposed with new COVID-19 cases, hospitalizations, and deaths per day in NYC. *New York State Department of Health. (2020, August 26). *COVID-19 testing*: *Antibody testing*. NY.gov. https://coronavirus.health.ny.gov/covid-19-testing.

In late December 2019, a viral pathogen causing severe respiratory problems was first detected in Wuhan, China (severe acute respiratory syndrome coronavirus 2; SARS-CoV2). SARS-CoV2 infections quickly spread globally, spurring multi-national travel restrictions; on March 11, 2020 the World Health Organization made a pandemic declaration for coronavirus disease 2019 (COVID-19). The greater metropolitan area of New York City (NYC) identified its first confirmed case of SARS-CoV2 viral infection on February 29, 2020 and within a month’s time, NYC emerged as one of the global epicenters of COVID-19 cases (Fig 1). Together with experiencing mass casualties (17,750 deaths in NYC by May 31, 2020), NYC residents were subjected to a 78-day “stay-at-home” order that confined all city inhabitants other than essential workers to their homes.

A body of literature [1] has documented an array of adverse psychological outcomes of pandemics and associated mass confinement, including elevated rates of stress, anxiety, depression and posttraumatic stress disorder (PTSD). Although research specific to COVID-19 remains limited [2], there is growing concern that the combination of COVID-19 outbreaks and the use of prolonged stay-at-home orders exact a significant toll on affected communities [3–5]. Indeed, the COVID-19 pandemic has generated unprecedented challenges to health, social and economic systems worldwide.

Evidence from general population studies suggests that young adults may be especially vulnerable to the psychological sequalae of a COVID-19 outbreak. Studies from China [3,6,7], Spain [4,8], India [9], and the United Kingdom [10] have indicated that student status and younger age are associated with heightened psychological distress and psychiatric impairment during the current COVID-19 pandemic. Some have suggested that the relative precariousness of young adults’ financial, professional, and social resources may be particularly sensitive to the upheaval caused by a major public health crisis [10–12].

As of June 2020, roughly 2.5 million NYC residents filed unemployment claims due to the state-mandated shutdown implemented between April and June 2020 [13]. Furthermore, preliminary evidence indicates that low income, older, and predominantly Black or Hispanic/Latinx communities within NYC share a disproportionate burden of the pandemic, including higher risk of infections, deaths and job losses, burdens that exacerbate prevailing health and economic disparities among under-resourced communities [14–18]. Moreover, differences in COVID-19 consequences have emerged between men and women, regardless of race or ethnicity. Although men face a higher risk of COVID-19 infection severity and death than women [19,20], women shoulder a disproportionate burden related to caring for sick loved ones, childcare and home schooling duties [21–24]. Additionally, more women than men report negative mental health impacts of the pandemic including higher worry, anxiety, depression, and stress [22]. Yet, given the rapidly changing environment of the current pandemic, much is still needed to be understood about how it has impacted the lives of many, particularly urban, student populations.

### Current study

The current study probed the consequences of the COVID-19 epidemic for an urban college population: students attending the City College of New York (CCNY) of the City University of New York (CUNY), a commuter college located in West Harlem, upper Manhattan. CCNY students come from highly diverse racial/ethnic backgrounds: Of the 15,465 students enrolled in 2019, the largest proportions of students identified as Hispanic/Latinx (36.9%), Asian (22.5%), Black (15.2%), or White (16.8%) [25]. The median parental income of CCNY students is $40,200, with 23% of families falling in the lowest income quintile of the local neighborhood [26].

Few studies have been conducted on the impact of COVID-19 on the college population, with most emerging from China. To the best of our knowledge, the study herein is the first to comprehensively examine the prevalence of specific COVID-19-related experiences and mental health functioning of US college students during prolonged mass confinement. Moreover, previous investigations were limited in scope, often focusing only on symptoms of generalized anxiety [11], psychological distress [10], or PTSD and depression [27]. Taken together, these studies suggest that COVID-19 is associated with elevated rates of stress, anxiety, depression, and, to a lesser extent, posttraumatic stress symptoms in affected college populations.

Unlike investigations that focus on a narrow subset of mental health outcomes, the current study sought to investigate a much broader range of pandemic-related personal and social impacts specifically: (a) current mental health functioning; (b) physical health problems of participants and their family members, (c) household makeup, living conditions, and family functioning, (d) employment and work life, (e) social interactions and social support, and (f) positive experiences. In particular, we aimed to (1) document the breadth of COVID-19 experiences in an ethnically and racially diverse college-aged sample; (2) explore potential associations between patterns of COVID-19 experiences and psychosocial health functioning during pandemic-related confinement, and (3) explore differences between women and men, and between different racial and ethnic groups, in COVID-19-related experiences and mental health functioning during the COVID-19 pandemic.

We were especially interested in probing these latter differences in our student population, given emerging evidence documenting the greater psychosocial burden of a major pandemic outbreak [3,6,11] on women [4,10,12] and racial/ethnic minorities [14–18]. With this study, we hope to contribute to the growing literature documenting COVID-19 impacts on the health of college-aged populations, and to inform decision-making regarding future directions to protect the well-being of student populations during the pandemic within academic institutions across the United States [28].

## Method

### Participants and procedure

This cross-sectional study used a convenience sampling approach and was approved by the Institutional Review Board of CUNY. Participants were recruited using school-wide broadcast emails and via the subject pool of CCNY. Students recruited via the subject pool were awarded course credit in exchange for completion of the survey. Those who enrolled through the broadcast email were entered into a raffle for two $500 gift cards. Interested students completed an online, forced-response format survey that was available from May 1 through May 31, 2020. A total of 1,507 responses were collected: 413 respondents started the survey, but did not complete it, and were therefore removed; 114 were removed because they either took multiple days to submit the survey (*n* = 30) or took less than 20 minutes to complete it (*n* = 30). Finally, 69 duplicates and two participants under the age of 18 were removed, thus yielding a final analytical sample of 909 individuals. Removed and retained participants were similar in terms of sex assigned at birth *χ*(1)^2^ = 0.54, *p* = .86, age *t*(1232) = 1.59, *p* = .11, year in school *χ*(6)^2^ = 8.15, *p* = .23, race/ethnicity *χ*(4)^2^ = 4.53, *p* = .34, sexual orientation *χ*(9)^2^ = 7.91, *p* = .54, international student status *χ*(1)^2^ = 0.26, *p* = .67, and residential location before *χ*(1)^2^ = 3.81, *p* = .05 or after Covid-19 *χ*(1)^2^ = 0.67, *p* = .41.

In order to assess respondents’ attention while taking the survey and potentially increase quality in responses [29], we embedded three attention check items in the survey, each of which had an obvious correct response (e.g., “Please choose the answer that matches the word Tiger” with answering options Lion, Bear, Tiger, and Whale). Almost all students (96%) failed none or only one attention check. We did not exclude any respondents based on their attention check results [30,31], but rather took these results as evidence that the vast majority of students included in the analyses were sufficiently attentive to the survey.

The final analytical sample was similar to the university’s student population [32] in terms of the percent who was between the ages of 18 and 21 years (*z* = 1.56, *p* = .12), international student (*z* = 0.32, *p* = .75), White (*z* = 0.82, *p* = .41), Black (*z* = 1.46, *p* = .14), or Hispanic/Latinx (*z* = 0.42, *p* = .68). Relative to the university student population, the sample had a slightly higher representation of females (*z* = 10.16, *p* < .001), Asian or Pacific Islander (*z* = 5.21, *p* < .001) and Multi-racial students (*z* = 5.19, *p* < .001). Study participants were on average 24.30 years old (SD = 6.64). The vast majority (87.8%) were undergraduate students and female (69.2%). Sixty-one participants (6.7%) indicated that they were international students. Additional characteristics are presented in Table 1. As shown, most participants were full-time students, and not in a relationship. Prior to the COVID-19 pandemic, the majority were living with a parent or guardian and were employed off-campus.

**Table 1:** Socio-demographic characteristics of the student survey sample (N = 909).

	N	%
**Sex (assigned at birth)**		
Female	629	69.2
Male	280	30.8
**College enrollment status**		
Full-time	750	82.5
Part-time	150	16.5
Other	9	1.0
**Race/Ethnicity**^a^		
White	160	17.6
Black	162	17.8
Hispanic/Latinx	344	37.8
Asian or Pacific Islander	251	27.6
Native American	9	1.0
Multiracial	56	6.2
Other	61	6.7
**Relationship status**		
Not in a relationship	537	59.1
In a relationship, not cohabiting	248	27.3
In a relationship, cohabiting	124	13.6
**Living situation before COVID-19**^b^		
Campus residence hall	40	4.4
Rental/apartment	317	34.9
Parent/Guardian’s home	552	60.7
**Employment status before COVID-19**^c^		
Not employed	342	37.6
Full-time on campus	8	0.9
Part-time on campus	84	9.2
Full-time off campus	151	16.6
Part-time off campus	324	35.6

^a^Multiple answers possible.

^b^Responses to the question “Where did you live before the COVID-19 campus closure (March 14th, 2020)?”.

^c^Responses to the question “Were you employed before the COVID-19 campus closure (March 14^th^, 2020)?”.

### Measures

**Sociodemographic information** (e.g., age, race, ethnicity, year in college, etc.) and COVID-19 related changes in students’ lives and activities (i.e., “How many times were you in contact with your friends in a typical week (outside of classes) [before/after] the COVID-19 campus closure?”) were obtained with a study-designed questionnaire.

**The Epidemic-Pandemic Impacts Inventory** (EPII, 92 items) [33] was used to explore pandemic-related experiences since the COVID-19 campus closure. The EPII is a novel inventory, developed to provide a comprehensive assessment of negative and positive pandemic-related experiences within the respondent’s household across major social and personal domains (Home Life, Work Life, Social Activities and Isolation, Education and Training, Emotional and Physical Health, and Positive Experiences). Each item contains a response set that includes an answer that pertains to the participant (Yes, Me), an answer that pertains to persons in the household (Yes, Person in home; except for items 42, 43, and 65), No, and Not applicable. To provide an overview of impacts of the COVID-19 pandemic on the lives of our students, we added the number of COVID-19 related experiences and impacts since the pandemic began within each domain (except Education and Training, which contains only two items) of the EPII to create a cumulative risk index. Preliminary findings from the EPII indicate that the risk index is both practical and statistically robust in predicting outcomes [33].

**The Depression, Anxiety and Stress Scales** (DASS, 42 statements) [34] was used to assess participants’ levels of stress, anxiety, and depressive symptoms. The DASS prompts respondents to indicate the degree to which they agree with statements relating to various symptoms of depression, anxiety and stress on a four-point scale (0: Never to 3: Almost always).

**The Patient Health Questionnaire** (PHQ-9, 9 items) [35] measured depressive symptomatology. PHQ-9 assesses the severity of depressive symptoms experienced over the past two weeks, ranging from 0 (Not at All) to 3 (Nearly Every Day), with a maximum score of 27. A PHQ score of >10 is indicative of probable depression [35].

**The General Anxiety Disorder (GAD)-7** (7 items) [36] assessed the severity of participants’ anxiety. The GAD-7 measures anxiety symptoms experienced over a two-week period, with response sets ranging from 0 (Not at All) to 3 (Nearly Every Day), with a maximum score of 21. A GAD-7 score of >10 is indicative of probable GAD [36].

**The Multidimensional Scale of Perceived Social Support** (MPSS, 12 items) [37] was employed to assess social support. The MPSS measures perceived adequacy of social support throughout three domains: family, friends and significant other. Respondents are asked to what extent they agree or disagree with a particular statement on a Likert scale ranging from 1 (Very Strongly Disagree) to 7 (Very Strongly Agree). A MPSS score of >5 suggests high perceived social support [38].

**The Primary Care PTSD Screen for DSM-5** (PC-PTSD-5, 5 items) [39] was used to assess for PTSD symptoms. The PC-PTSD-5 yields a response range from 0–5, with higher scores reflecting a higher likelihood of a PTSD diagnosis. A cut-point score of 4 on the PC-PTSD-5 has been recommended for a probable PTSD diagnosis [39].

### Data analysis

COVID-19 related impacts experienced by the students are presented descriptively. Comparisons based on sex and racial/ethnic group were conducted using independent-samples t-tests and one-way analyses of variance (ANOVA), respectively. Welch’s statistic is reported in cases where the assumption of homogeneity of variance was not met. In case of significant ANOVAs, Tukey pairwise comparisons identified where the group differences lied. Multiple regressions were conducted to predict each mental health outcome (DASS Stress, DASS Anxiety, DASS Depression, PHQ-9, GAD-7, and PC-PTSD-5) from the different EPII risk indices. Distributional properties of EPII risk indices were examined for non-normality. All indices fell within the acceptable range for skewness (+/- 2) and kurtosis (+/- 7) [40], except for Infection History (skewness = 3.46; kurtosis = 19.40).

In addition, we compared three groups of participants impacted by COVID-19 infection regarding their psychological functioning (DASS, PHQ-9, GAD-7) and their perceived social support (PSS) using multivariate analysis of variance (MANCOVA) with age, sex and racial/ethnic group as covariates. Group 1 comprised students who had experienced a COVID-19 death in their home or of a close friend/family member and who may or may not have a positive infection history themselves (*N* = 155); Group 2 comprised students with a positive infection history but no experience of COVID-19-related death (*N* = 149); and Group 3 were those who had a negative infection history and had not experienced the death of someone close to them (*N* = 605). Given the forced response format of the online survey, there were no missing data. However, Q-Q plots showed some deviation of residuals from the normal distribution, indicating potential problems with multivariate normality, which is why we interpreted Pillai’s Trace for multivariate tests.

## Results

### COVID-19 related changes in the students’ life

Around 15% (*n* = 139) of the sample indicated that they had relocated since the COVID-19 campus closure on March 14, 2020. Half of those (*n* = 70, 50.4% of those who had relocated) indicated that they relocated to their parents’ house or the home of a relative, eleven (7.9%) had moved in with their partner, and seven (5.0%) had moved to a different apartment. Twenty-eight students (20.1% of those who relocated) indicated that they had left the state or the country.

Students reported that they had been in contact with their friends on average 4.73 times (SD = 5.33) in a typical week (outside of classes) before the COVID-19 campus closure and 3.76 (SD = 5.89) after the closure, which represents a significant reduction in contacts from pre- to post-campus closure, *t*(898) = 5.28, *p* < .001, *d* = 0.165. In addition, students indicated that in-person contacts were considerably reduced: 45.0% of contacts before the closure were in-person and only 8.5% after the closure.

Around 42% of students (*n* = 383) indicated that their employment situation had changed due to COVID-19. The vast majority of those were no longer employed (n = 299, 78.1%), 66 (17.2%) were working fewer hours/week, and 15 (3.9%) were working more hours/week.

### COVID-19 related impacts experienced by the students (EPII)

Table 2 contains the number/percentage of students in each gender and racial/ethnic category who experienced at least one impact in each EPII domain and the average number of experiences among those who endorsed at least one impact in the respective domain. Table 3 reports on the three most common experiences and impacts (including the percentage of women and men who endorsed these three events per domain) for each domain. Additional relevant information is reported below.

**Table 2:** COVID-19 impacts reported by the surveyed student sample (N = 909), and by sex and racial/ethnic group.

EPII domain (number of items/impacts)	Total Sample (N = 909)	Women (*N* = 629)	Men (*N* = 280)	White^b^ (*N* = 124)	Black^b^ (*N* = 128)	Asian^b^ (*N* = 249)	Hispanic/Latinx^b^ (*N* = 344)
**Work & Employment (11)**							
N (%) of students who endorsed ≥1 impact	720 (79.0)	522 (82.9)	198 (70.5)	94 (75.8)	103 (80.5)	186 (74.7)	286 (82.7)
Mean (SD) number of reported impacts^a^	2.79 (1.73)	2.81 (1.72)	2.71 (2.76)	2.56 (1.69)	2.59 (1.58)	2.88 (1.78)	1.76 (1.69)
**Home Life (13)**							
N (%) of students who endorsed ≥1 impact	537 (58.9)	405 (64.3)	132 (47.0)	70 (56.5)	75 (58.6)	144 (57.8)	205 (59.2)
Mean (SD) number of reported impacts^a^	2.18 (1.46)	2.26 (1.48)	1.91 (1.35)	1.9 (1.23)	2.37 (1.59)	2.2 (1.40)	2.14 (1.34)
**Social Activities (10)**							
N (%) of students who endorsed ≥1 impact	885 (97.1)	620 (98.4)	265 (94.3)	121 (97.6)	125 (97.7)	239 (96.0)	338 (97.7)
Mean (SD) number of reported impacts^a^	5.00 (2.11)	5.06 (2.08)	4.71 (2.18)	4.93 (1.96)	5.01 (2.13)	5.05 (2.19)	4.77 (2.04)
**Economic (5)**							
N (%) of students who endorsed ≥1 impact	527 (57.8)	362 (57.5)	165 (58.7)	65 (52.4)	69 (53.9)	154 (61.8)	208 (60.1)
Mean (SD) number of reported impacts^a^	1.82 (0.97)	1.82 (0.95)	1.81 (1.00)	1.49 (0.82)	1.86 (0.97)	1.99 (1.05)	1.75 (0.89)
**Emotional Health & Well-being (8)**							
N (%) of students who endorsed ≥1 impact	853 (93.6)	602 (95.6)	251 (89.3)	115 (92.7)	121 (94.5)	228 (91.6)	331 (95.7)
Mean (SD) number of reported impacts^a^	3.32 (1.58)	3.39 (1.57)	3.18 (1.59)	3.39 (1.52)	3.22 (1.64)	3.31 (1.65)	3.28 (1.50)
**Physical Health Problems (8)**							
N (%) of students who endorsed ≥1 impact	844 (92.6)	595 (94.4)	249 (88.6)	118 (95.2)	118 (92.2)	226 (90.8)	321 (92.8)
Mean (SD) number of reported impacts^a^	3.18 (1.21)	3.14 (1.29)	3.20 (1.17)	2.89 (1.13)	3.26 (1.24)	3.37 (1.26)	3.13 (1.12)
**Physical Distancing & Quarantine (8)**							
N (%) of students who endorsed ≥1 impact	650 (71.4)	465 (73.8)	185 (65.8)	85 (68.5)	76 (59.4)	170 (68.3)	268 (77.5)
Mean (SD) number of reported impacts^a^	2.58 (1.51)	2.59 (1.49)	2.56 (1.55)	2.64 (1.50)	2.28 (1.48)	2.76 (1.59)	2.46 (1.39)
**Infection History (8)**							
N (%) of students who endorsed ≥1 impact	304 (33.4)	220 (34.9)	84 (29.9)	40 (32.3)	35 (27.3)	61 (24.5)	141 (40.8)
Mean (SD) number of reported impacts^a^	1.40 (0.79)	1.38 (0.81)	1.44 (0.75)	1.25 (0.54)	1.29 (0.62)	1.51 (0.74)	1.35 (0.72)
**Positive Change (19)**							
N (%) of students who endorsed ≥1 impact	876 (96.2)	615 (97.6)	261 (92.9)	119 (96.0)	126 (98.4)	233 (93.6)	338 (97.7)
Mean (SD) number of reported impacts^a^	7.03 (3.60)	7.20 (3.59)	6.63 (3.59)	6.46 (3.32)	7.39 (4.08)	6.80 (3.71)	7.36 (3.33)

^a^Among those who endorsed at least 1 impact in the respective domain;

^b^In this analysis, all participants were assigned to one distinct category (i.e., students endorsing Hispanic/Latinx and another racial/ethnic category [White, Black, Asian, Native American, Multiracial and Other] were categorized as Hispanic/Latinx; for this reason, percentages may differ from Table 1). Given the small percentages of students who were categorized as Native American, Multiracial or Other, only the White, Black, Asian, and Hispanic/Latinx ethnic/racial groups were included in the comparison.

**Table 3:** Most common COVID-19 impacts in each EPII domain by the surveyed student sample (N = 909) and by sex.

		Most commonly endorsed COVID-19 impacts	% of Sample	% Women	% Men
**EPII Domain**	**Infection History**	Having had symptoms of COVID-19	20.3	20.3	20.3
		Death of a close friend or family member from COVID-19	17.0	18.1	14.6
		Current symptoms of COVID-19	4.1	3.7	5.0
	**Work & Employment**	Difficulties in transitioning to working from home	39.5	43.4	31.0
		Increase in workload or work responsibilities	33.5	36.7	26.3
		Reduction in work hours or having been furloughed	31.5	32.1	30.2
	**Education & Training**	Adult unable to go to school or training for weeks or had to withdraw	30.0	29.5	31.0
		Child in the home who could not go to school	25.5	26.0	24.2
	**Home Life**	Increases in verbal arguments or conflicts with adults (other than partner) in the home	28.9	32.1	21.7
		Spending a lot more time caring for a family member	23.4	25.6	18.5
		Having to take over teaching or instructing a child	16.7	20.3	8.5
	**Social Activities**	Inability to do enjoyable activities or hobbies	83.3	85.2	79.0
		Cancelled or restricted family celebrations	72.2	75.9	64.1
		Separation from family or close friends	71.9	76.5	61.6
	**Economic**	Difficulty getting places due to less access to public transportation or concerns about safety	39.8	39.7	40.2
		Unable to pay important bills like rent or utilities	26.1	25.9	26.7
		Unable to get enough food or healthy food	23.7	23.5	24.2
	**Emotional Health & Well-Being**	Increased amount of time spent on screens and devices	87.9	88.7	86.1
		Increase in sleep problems or poor sleep quality	72.6	75.1	66.9
		Increase in mental health problems or symptoms	67.7	72.5	56.9
	**Physical Distancing & Quarantine**	Increased amount of time sitting down or being sedentary	87.5	89.5	82.9
		Less physical activity or exercise	79.3	79.2	79.4
		Overeating or eating more unhealthy foods	63.0	67.6	52.7
	**Positive Change**	Increased appreciation of things usually taken for granted	76.6	81.4	65.8
		More quality time with family or friends in person or from a distance	72.9	75.4	67.3
		Increased attention paid to personal health	65.8	66.8	63.3

#### Infection history

Nine students reported that they had been tested positive and currently had the disease (1.0%; eight of those were women), and 16 reported that they had tested positive but no longer had the disease (1.8%; twelve of those were women). Seventeen students had received medical treatment due to severe symptoms of COVID-19 (1.9%; thirteen of those were women); 4 reported a hospital stay due to COVID-19 (0.4%; all of these were women); two students indicated that someone had died of COVID-19 while in their home (both of these were women). There was no sex difference in the average number of experiences in this domain, *t*(301) = 0.58, *p* = .564, and no difference between ethnic/racial groups, *F*(3,272) = 1.40, *p* = .243.

#### Work and employment

Twenty-nine percent of students indicated that they had been laid off their job or had to close their own business (30.0% of women, 27.4% of men); 25.0% reported that they had a hard time doing their job well because they needed to take care of people in their home (27.5% of women, 19.6% of men); and 10.2% reported that they had to continue working despite being in close contact with people who might be infected (11.1% of women, 8.2% of men). There was no sex difference in the average number of experiences in this domain, *t*(716) = -.617, *p* = .538, nor was there a difference between ethnic/racial groups, *F*(3,663) = 1.10, *p* = .347.

#### Home life

In accordance with our demographic data, 13.4% of respondents indicated that they had to move or relocate since the COVID-19 pandemic began (14.9% of women, 10.0% of men), and four students reported that they had become homeless (all women). Women had experienced significantly more impacts in this domain than men, *t*(534) = -2.43, *p* = .015, *d* = 0.247; however, there was no significant difference between racial/ethnic groups in the number of experienced impacts, *F*(3,194.56) = 1.41, *p* = .241.

#### Social activities

Twenty-seven percent reported that they were unable to be with a close family member in critical condition (27.5% of women, 26.3% of men); and 23.8% were unable to attend a funeral or religious service for a family member or friend who died (24.9% of women, 21.0% of men). Women had experienced significantly more impacts in this domain than men, *t*(881) = -2.19, *p* = .029, *d* = 0.160; however, there was no significant difference between racial/ethnic groups in the number of experienced impacts, *F*(3,817) = .852, *p* = .465.

#### Economic

There was no sex difference in the average number of experiences in this domain, *t*(523) = -.062, *p* = .951; however, there was a significant difference between ethnic/racial groups, *F*(3,484) = 4.238, *p* = .006, partial η^2^ = .026. Post-hoc tests revealed that Asian students had experienced a significantly higher number of impacts on average compared with White participants, *p* = .004.

#### Emotional health and well-being

More than 20% of students (n = 194) indicated that they were unable to access mental health treatment or therapy (22.4% of women, 18.9% of men); 15.7% reported increased sleep difficulties in their child (17.1% of women, 12.5% of men); and 14.5% reported increased behavioral or emotional problems in their child (16.2% of women, 10.7% of men). There was no sex difference in the average number of experiences in this domain, *t*(849) = -1.69, *p* = .091, and no significant difference between racial/ethnic groups, *F*(3,789) = .233, *p* = .873.

#### Physical health problems

Only a small number of students reported that they were unable to access medical care for a serious condition (2.9%; 2.1% of women, 4.6% of men). There was no sex difference in the average number of experiences in this domain, *t*(840) = -0.69, *p* = .489; however, there was a significant difference between ethnic/racial groups, *F*(3,315.73) = 4.72, *p* = .003, partial η^2^ = .018. Post-hoc tests revealed that Asian students had experienced a significantly higher number of impacts compared with White participants, *p* = .002.

#### Physical distancing and quarantine

There was no sex difference in the average number of experiences in this domain, *t*(646) = -0.15, *p* = .880, and no difference between ethnic/racial groups, *F*(3,593) = 2.51, *p* = .058.

#### Positive change

Women reported a significantly higher number of positive impacts when compared to men, *t*(872) = -2.18, *p* = .029, *d* = 0.162. In addition, there was a significant difference between ethnic/racial groups, *F*(3,324.57) = 2.93, *p* = .034, partial η^2^ = .010. Post-hoc tests revealed that Hispanic/Latinx students had a statistical trend towards having experienced a higher number of positive impacts when compared to White participants, *p* = .078.

### Students’ mental health functioning during the COVID-19 pandemic

Correlations between the mental health outcomes are presented in S1 Table. Table 4 summarizes students’ mental health functioning in terms of mean stress (DASS stress subscale), anxiety (DASS anxiety subscale; GAD-7), and depression (DASS depression subscale; PHQ-9) scores in the past two weeks, lifetime PTSD (PC-PTSD 5), and PSS, together with the number of students who exceeded the cut-offs on each scale (if applicable). Close to 90% of students exceeded the cut-off score for the PHQ, indicating severe symptoms of depression in the past 2 weeks, and two-thirds reported severe symptoms of anxiety (GAD) in the same time period. Roughly five percent of our sample had a PC-PTSD score indicative of a possible PTSD diagnosis (lifetime). At the same time, more than 60% of the sample perceived that their social support was high (Table 4).

**Table 4:** Mental health of the surveyed student sample (N = 909).

	DASS Stress	DASS Anxiety	DASS Depression	PHQ-9	GAD-7	PC-PTSD 5	PSS Total
**Mean score (SD)**	12.93 (4.80)	10.95 (4.11)	13.21 (5.37)	18.42 (6.56)	14.04 (5.95)	2.73 (1.42)	5.26 (1.32)
**Exceeded cut-off, *N* (%)**	-	-	-	817 (89.9)	599 (65.9)	43 (4.7)	554 (60.9)

Multiple regressions were conducted to predict each mental health outcome (DASS Stress, DASS Anxiety, DASS Depression, PHQ-9, GAD-7, and PC-PTSD-5) from the different EPII risk indices. The risk indices significantly predicted anxiety as measured by the DASS subscale, *F*(9, 112) = 2.28, *p* = .022, *R*^*2*^ = .16, and the GAD, *F*(9, 112) = 3.37, *p* < .001, *R*^*2*^ = .21. However, none of the risk indices added statistically significantly to the prediction of the DASS anxiety score, all *p* > .05. As for the GAD score, only the Emotional Health & Well-being (*p* = .002) and the Positive Change (*p* = .042) indices added significantly to the prediction. In addition, the risk indices significantly predicted depression as measured by the DASS subscale, *F*(9, 112) = 2.48, *p* = .013, *R*^*2*^ = .17, and the PHQ, *F*(9, 112) = 2.29, *p* = .021, *R*^*2*^ = .16. The Emotional Health & Well-being index added significantly to the prediction of both the DASS depression score, *p* = .006, and the PHQ score, *p* = .026. The Positive Change index added to the prediction of the DASS depression score, *p* = .025, but not the PHQ outcome. Neither stress, measured with the DASS subscale, nor PTSD were statistically significantly predicted by the risk indices. Multicollinearity was not problematic as the tolerance was well above.2 and the variance inflation factor (VIF) was well below 10 for all variables included in the analyses [40].

A statistically significant difference was found among the three infection history groups (Group 1: experience of COVID-19 related death of a person close to them; Group 2: positive infection history but no experience of COVID-19 related death; Group 3: negative infection history and no experience of COVID-19 related death) on the combined dependent variables (DASS subscales, PHQ, GAD and PSS) after controlling for age, sex and racial/ethnic group, *F*(12,1796) = 5.27, *p* < .001, Pillai’s Trace = .068, partial η^2^ = .034. Univariate analysis indicated that there was no difference between the three groups in perceived social support. Pairwise contrast revealed that Group 1 had the highest scores in all remaining scales (DASS subscales, PHQ, GAD), and all comparisons with Group 3 reached statistical significance, *p* < .001. Moreover, Group 2 had significantly higher scores compared to Group 3 in the DASS stress subscale, *p* = .017, and anxiety subscale, *p* = .037, as well as the PHQ, *p* = .006.

Sex was the only statistically significant covariate in the model, *F*(6, 897) = 3.94, *p* < .001, Pillai’s Trace = .026, partial η^2^ = .026. Univariate analysis indicated that women had significantly higher scores compared with men in DASS stress, *t*(907) = -3.844, *p* < .001, *d* = 0.277, anxiety, *t*(907) = -2.90, *p* = .004, *d* = 0.209, and depression subscale scores, *t*(907) = -2.17, *p* = .030, *d* = 0.154. In addition, women had higher scores in the PHQ, *t*(907) = -2.60, *p* = .010, *d* = 0.188, and the GAD, *t*(907) = -3.23, *p* = .001, *d* = 0.234, and had higher perceived social support, *t*(907) = -2.32, *p* = .020, *d* = 0.163. There were no sex differences in PC-PTSD scores, *t*(260) = -0.88, *p* = .378.

## Discussion

### Experiences and consequences related to COVID-19

In the immediate aftermath of NYC’s peak SARS-COV2 infection rate and six weeks into a shelter-at-home order, the current study sought to chart COVID-19 experiences and their impact upon psychosocial functioning in a racially and ethnically diverse sample of NYC college students. Our findings confirm the extent to which NYC represented the global epicenter of COVID-19 in the spring of 2020 and the pandemic’s fundamental upheaval of daily life. Specifically, they reveal that the COVID-19 pandemic affected all dimensions of the CCNY students’ quality of life during the physical lockdown of NYC, many of which extended beyond its effects on mental health functioning. Strikingly, students directly faced the health consequences and mortality of COVID-19: 20.3% reported symptoms of the virus and 17% reported the death of a family member or close friend from the virus. These results align closely with those from a study of NYC residents (*n* = 286) conducted during the same period of our survey (May 5–12, 2020): 42% of respondents reported knowing someone who had tested positive for COVID-19, and 23.1% reported knowing someone who died from COVID-19 [41]. Many of our respondents reported virus symptoms without testing, perhaps due to ongoing difficulties in obtaining testing. During the week of May 5–12, the NYC Department of Health reported that only 110,821 tests had been administered throughout the metropolitan area [42].

Moreover, CCNY students confronted to an unparalleled degree the health and mortality burden of the disease directly in their homes. In prior research on college-aged adults during COVID-19, prevalence rates of knowing someone infected with COVID-19 or who had died from COVID-19 hover below 1% [11,27]. In the hardest-hit province of Hubei in China, study respondents with suspected or confirmed cases of COVID were reported to account for 1% of the total sample [12]. Thus, to the best of our knowledge, the current findings document the highest rate of COVID-19 infection and exposure to death in sampled populations to date.

Our findings further indicate that women, irrespective of race or ethnicity, reported higher disruptions related to COVID-19 than men. Our findings fall in line with recent evidence from the U.S. and elsewhere highlighting a gender gap in negative impacts from the pandemic [21–24]. In this vein, the findings highlight the utility of social programs seeking to mitigate the added burden faced by women during the current pandemic and the need to restructure social services for women in anticipation of future pandemics. At the same time, we found that women were more likely than men to report positive changes since the pandemic began. We also found that Hispanic/Latinx participants reported more positive experiences than White participants. These findings are significant in suggesting that, despite added burden, both women and Hispanic/Latinx students may be more resilient and adaptable about their circumstances than men or White students. Future research should investigate whether resilience differs across sex and race/ethnicity and what protective role, if any, said resilience plays against mental health consequences during multiple stages of the pandemic. Findings in this direction can inform the creation of intervention programs seeking to improve resilience in student populations, potentially mitigating of high risk in as much as to mitigate the pandemic’s long-term effects.

Differences were also observed between Asian students when compared to White students in economic and physical health impacts of COVID-19. Given the connection between eating healthy, exercising and long-term health [43,44], our findings highlight the value and need for social and university-supported programs to reduce food insecurity and income inequality, and improve access to healthy foods and promote physical activity, particularly among under-resourced students.

### Psychological functioning during lockdown

Our findings pertaining to the mental health during this prolonged confinement are concerning. Reports of clinically significant levels of stress, anxiety, and depression were present at exceedingly higher rates than prior published data on COVID-19-affected populations, which have ranged from 15% to 48% for depression and 6 to 51% for anxiety [2]. One possible reason for the divergence from prior COVID-19 work is that the pandemic-related exposures and impacts reported by our sample appear to differ by an order of magnitude, most notably in infection and mortality exposure. Relatedly, the current study sampled individuals who had already experienced *at least* six weeks of prescribed physical confinement, per a New York State “shelter-at-home” mandate. Pandemic-confined samples studied to date had significantly briefer periods of home isolation, and to the best of our knowledge, none as long as the present sample. Thus, it may be of limited value to attempt direct comparisons between the mental health functioning of published COVID-19 and the current study. Future research on samples with comparable rates of COVID-19 exposure and impacts will be necessary to contextualize the significance and relationship of these mental health findings to the pandemic.

Of note, the rate of a potential PTSD diagnosis in our sample is in line with prevalence rates in the US general population [45]. Thus, perhaps the severe levels of stress, anxiety and depression—which all far exceed population 12-month prevalence rates of the corresponding disorders (e.g., major depressive disorder: 5.3%; anxiety disorders: 0.1% [agoraphobia] to 7.1% [specific phobia] [46])–might be passing, reactive states rather than indicators of manifesting disorders. In addition, perceived social support, which is an important protective factor against the negative effects of adverse events on mental health [47], was robust in our sample, perhaps because the vast majority of these students lived with their families during lockdown. Nonetheless, sources of social support may diminish during prolonged periods of disrupted everyday life—particularly in locations where COVID-19 infection rates remain high and distancing and confinement measures continue or are re-introduced. Thus, the mental health and social support of college-aged populations should continue to be monitored over the course of the pandemic and additional supporting structures implemented if the need arises.

Our findings regarding women’s mental health burden align with other COVID-19 studies that have found higher rates of anxiety, depression, and stress using similar well-validated, self-report measures [4,10,12]. These sex differences in psychiatric symptomatology were found in spite of women and men reporting similar cumulative risk index levels of COVID-19 emotional health and well-being experiences. This apparent discrepancy is likely due in part to the difficulty of comparing an event-inventory scale such as the EPII to stand-alone discrete assessments of psychological distress. Future research is tasked with examining the extent to which women attribute mental health burdens to specific stressors such as COVID-19 and its connection to psychological functioning.

Associations between COVID-19 impact and mental health functioning were found across almost all pandemic experience and consequence domains. This aligns with prior research identifying relationships between having a relative infected by COVID-19 [6,48], residing in a severely affected area [27,48,49], perceiving more daily impacts [5,11], and worse psychological functioning. Similarly, our finding that positive changes in response to COVID-19 were associated with lower levels of anxiety and depression converges with COVID-19-specific research on potential sources of resilience including good family functioning [10] and positive reappraisal [50].

Unsurprisingly, those exposed to the most likely traumatic aspect of the pandemic—the death of a close or friend or relative—reported significantly higher rates of depression, stress, and anxiety when compared to students with infection histories that did not involve a death. This finding underscores the importance of developing and delivering resources to bereaved and grieving populations. It is likely that COVID-19’s restriction on collective rituals of mourning—e.g., saying goodbye, wakes, funerals, and memorials—has exacerbated the tragic dimensions of losing a loved one to a pandemic. Indeed, studies have shown that the absence of traditional mourning practices [51] and physical support [52] increase the severity of prolonged grief symptoms in bereaved populations. Unresolved loss, it appears, may be one of the lasting psychological consequences of COVID-19, which may require clinicians to expand traditional bereavement support.

### Limitations

This study has several limitations. First, the study was composed of a convenience sample and is thus subject to the risk of sampling bias, where college students with the most distress may have been the most likely to participate and complete the study. The possibility of this type of sampling bias suggests that prevalence rates must be interpreted with caution. Notably, the sample’s socio-demographic characteristics were diverse and map on well to the distribution of students attending CCNY [25]. Although the sample may have overrepresented one portion of the US student population, the significant relations found between participant experiences, demographic characteristics, and mental health functioning remain valid. Second, the study’s cross-sectional design prohibits any causal conclusions about the contribution of COVID-19-related experiences and impacts on students’ mental health. Although our data validation methods did not indicate that lack of data quality is of concern, the online self-report format raises concerns regarding the potential for desirability bias influencing our findings. Lastly, we note that, despite conforming to demographic standards of the National Institute of Health (NIH), racial/ethnic classifications in the current study were somewhat arbitrary [53] and findings on racial/ethnic group differences should be interpreted cautiously. Further exploration of contextual factors, such as socioeconomic variables, are critical for understanding the differential associations between the racial/ethnic groups examined and COVID-19.

In spite of these limitations, this study uniquely adds to the literature by comprehensively exploring mental health correlates of COVID-19 impacts on the daily life of a diverse student sample during the early stages of the pandemic in its U.S. epicenter during the time frame of this investigation. In so doing, it establishes a roadmap for future research exploring the mental health implications of this and other pandemics among diverse populations. Given the rapidly-changing and stressful environment that the COVID-19 has generated, coupled with widening health, social and economic inequalities, the current study underscores the importance of exploring the long-term consequences of the pandemic and identifying factors that enhance resilience in diverse populations [54].

### Conclusion

The current study explored the experience and impact of the unprecedented stressors of a COVID-19 outbreak—exposure to possible contagion of a life-threatening pathogen and a 78-day government-mandated home confinement—on a diverse NYC college sample. We found significant disruptions in the daily lives of the sampled students, in line with the city’s status as one of COVID-19’s highest-impact zones, coupled with high psychological distress. Our findings provide a benchmark for future studies of student populations facing COVID-19 and its aftermath and may provide preliminary guidance on how best to protect students’ well-being during these unprecedented times.

## Supporting information

S1 Table Means, standard deviations, and correlations with 95% confidence intervals of mental health measures [Depression, Anxiety and Stress Scales (DASS), Patient Health Questionnaire (PHQ-9), General Anxiety Disorder (GAD-7), and Primary Care PTSD Screen for DSM-5 (PC-PTSD-5)]. (DOCX)

S1 File Demographics and college activities survey questions. (DOCX)

## References

[1] Brooks SK, Webster RK, Smith LE, Woodland L, Wessely S, Greenberg N, et al. The psychological impact of quarantine and how to reduce it: rapid review of the evidence. The Lancet. 2020. 10.1016/S0140-6736(20)30460-8 32112714 PMC7158942

[2] Xiong J, Lipsitz O, Nasri F, Lui LMW, Gill H, Phan L, et al. Impact of COVID-19 pandemic on mental health in the general population: A systematic review. J Affect Disord. 2020;277: 55–64. 10.1016/j.jad.2020.08.001 32799105 PMC7413844

[3] Huang Y, Zhao N. Generalized anxiety disorder, depressive symptoms and sleep quality during COVID-19 outbreak in China: a web-based cross-sectional survey. Psychiatry Res. 2020. 10.1016/j.psychres.2020.112954 32325383 PMC7152913

[4] Rodríguez-Rey R, Garrido-Hernansaiz H, Collado S. Psychological Impact and Associated Factors During the Initial Stage of the Coronavirus (COVID-19) Pandemic Among the General Population in Spain. Front Psychol. 2020;11. 10.3389/fpsyg.2020.01540 32655463 PMC7325630

[5] Tull MT, Edmonds KA, Scamaldo KM, Richmond JR, Rose JP, Gratz KL. Psychological Outcomes Associated with Stay-at-Home Orders and the Perceived Impact of COVID-19 on Daily Life. Psychiatry Res. 2020. 10.1016/j.psychres.2020.113098 32434092 PMC7252159

[6] Wang ZH, Yang HL, Yang YQ, Liu D, Li ZH, Zhang XR, et al. Prevalence of anxiety and depression symptom, and the demands for psychological knowledge and interventions in college students during COVID-19 epidemic: A large cross-sectional study. J Affect Disord. 2020. 10.1016/j.jad.2020.06.034 32734907 PMC7330560

[7] Peng M, Mo B, Liu Y, Xu M, Song X, Liu L, et al. Prevalence, risk factors and clinical correlates of depression in quarantined population during the COVID-19 outbreak. J Affect Disord. 2020. 10.1016/j.jad.2020.06.035 32658813 PMC7330582

[8] González-Sanguino C, Ausín B, Castellanos MÁ, Saiz J, López-Gómez A, Ugidos C, et al. Mental health consequences during the initial stage of the 2020 Coronavirus pandemic (COVID-19) in Spain. Brain Behav Immun. 2020. 10.1016/j.bbi.2020.05.040 32405150 PMC7219372

[9] Varshney M, Parel JT, Raizada N, Sarin SK. Initial psychological impact of COVID-19 and its correlates in Indian Community: An online (FEEL-COVID) survey. PLoS One. 2020. 10.1371/journal.pone.0233874 32470088 PMC7259495

[10] Li Y, Wang Y, Jiang J, Valdimarsdóttir UA, Fall K, Fang F, et al. Psychological distress among health professional students during the COVID-19 outbreak. Psychol Med. 2020. 10.1017/S0033291720001555 32389148 PMC7225209

[11] Cao W, Fang Z, Hou G, Han M, Xu X, Dong J, et al. The psychological impact of the COVID-19 epidemic on college students in China. Psychiatry Res. 2020. 10.1016/j.psychres.2020.112934 32229390 PMC7102633

[12] Liu N, Zhang F, Wei C, Jia Y, Shang Z, Sun L, et al. Prevalence and predictors of PTSS during COVID-19 outbreak in China hardest-hit areas: Gender differences matter. Psychiatry Res. 2020. 10.1016/j.psychres.2020.112921 32240896 PMC7102622

[13] Diana C. The economic impact of Covid-19 in New York state by the numbers. 2020 [cited 29 Jul 2020]. https://www.bizjournals.com/albany/news/2020/06/08/new-york-economic-impact-of-covid-19.html.

[14] Chow N, Fleming-Dutra K, Gierke R, Hall A, Hughes M, Pilishvili T, et al. Preliminary Estimates of the Prevalence of Selected Underlying Health Conditions Among Patients with Coronavirus Disease 2019—United States, February 12–March 28, 2020. MMWR Morb Mortal Wkly Rep. 2020;69: 382–386. 10.15585/mmwr.mm6913e2 32240123 PMC7119513

[15] Dobkin J, Gottehrer-Cohen Z. The very unequal impact of COVID-19 on NYC neighborhoods. 2020 [cited 29 Jul 2020]. https://gothamist.com/news/very-unequal-impact-covid-19-nyc-neighborhoods.

[16] New York City Health Department. Age adjusted rate of fatal lab confirmed COVID-19 cases per 100,000 by race/ethnicity group. 2020 [cited 17 Jul 2020]. https://www1.nyc.gov/assets/doh/downloads/pdf/imm/covid-19-deaths-race-ethnicity-04082020-1.pdf.

[17] Pew Research Center. About half of lower-income Americans report household job or wage loss due to COVID-19. 2020 [cited 30 Jul 2020]. https://www.pewsocialtrends.org/2020/04/21/about-half-of-lower-income-americans-report-household-job-or-wage-loss-due-to-covid-19/psdt_04-21-20_covidimpact-00-7/.

[18] Webb Hooper M, Nápoles AM, Pérez-Stable EJ. COVID-19 and Racial/Ethnic Disparities. JAMA. 2020;323: 2466. 10.1001/jama.2020.8598 32391864 PMC9310097

[19] Jin J-M, Bai P, He W, Wu F, Liu X-F, Han D-M, et al. Gender Differences in Patients With COVID-19: Focus on Severity and Mortality. Front Public Heal. 2020;8. 10.3389/fpubh.2020.00152 32411652 PMC7201103

[20] Stokes EK, Zambrano LD, Anderson KN, Marder EP, Raz KM, El Burai Felix S, et al. Coronavirus Disease 2019 Case Surveillance—United States, January 22–May 30, 2020. MMWR Morb Mortal Wkly Rep. 2020. 10.15585/mmwr.mm6924e2 32555134 PMC7302472

[21] Azcona G, Bhatt A, Love K. Ipsos survey confirms that COVID-19 is intensifying women’s workload at home. 2020. https://data.unwomen.org/features/ipsos-survey-confirms-covid-19-intensifying-womens-workload-home.

[22] Frederiksen B, Gomez I, Salganicoff A, Ranji U. Coronavirus: A look at gender differences in awareness and actions. KFF Coronavirus Poll: March 2020. 2020. https://www.kff.org/coronavirus-covid-19/issue-brief/coronavirus-a-look-at-gender-differences-in-awareness-and-actions/.

[23] Hamel L, Salganicoff A. Is there a widening gender gap in coronavirus stress? KFF Coronavirus Poll: March 2020. 2020. https://www.kff.org/coronavirus-policy-watch/is-there-widening-gender-gap-in-coronavirus-stress/.

[24] Hupkau C, Petrongolo B. COVID-19 and gender gaps: Latest evidence and lessons from the UK. 2020. https://voxeu.org/article/covid-19-and-gender-gaps-latest-evidence-and-lessons-uk.

[25] CCNY Office of Institutional Research. INSTITUTIONAL RESEARCH. 2019 [cited 12 Aug 2020]. https://www.ccny.cuny.edu/institutionalresearch.

[26] Chetty R, Friedman JN, Saez E, Turner N, Yagan D. Mobility Report Cards: The Role of Colleges in Intergenerational Mobility. Natl Bur Econ Res Work Pap. 2017.

[27] Tang W, Hu T, Hu B, Jin C, Wang G, Xie C, et al. Prevalence and correlates of PTSD and depressive symptoms one month after the outbreak of the COVID-19 epidemic in a sample of home-quarantined Chinese university students. J Affect Disord. 2020;274: 1–7. 10.1016/j.jad.2020.05.009 32405111 PMC7217769

[28] Madhusoodanan J. University reopening plans under fire. Science. 2020. 10.1126/science.369.6502.359 32703857

[29] Shamon H, Berning C. Attention check items and instructions in online surveys with incentivized and non-incentivizedquality?Samples: Boon or bane for data. Surv Res Methods. 2020;14: 55–77. 10.18148/srm/2020.v14i1.7374

[30] Gummer T, Roßmann J, Silber H. Using Instructed Response Items as Attention Checks in Web Surveys: Properties and Implementation. Sociol Methods Res. 2018.

[31] Anduiza E, Galais C. Answering without reading: IMCs and strong satisficing in online surveys. Int J Public Opin Res. 2017. 10.1093/ijpor/edw007

[32] CCNY Office of Institutional Research. City Facts Fall 2018. New York; 2019.

[33] Grasso D, Briggs-Gowan M, Carter A, Goldstein B, Ford J. A Person-Centered Approach to ProfilingCOVID-Related Experiences in the United States: Preliminary Findings from the Epidemic-Pandemic Impacts Inventory (EPII). PsyArXiv. 2020. 10.31234/osf.io/v36hj

[34] Antony MM, Cox BJ, Enns MW, Bieling PJ, Swinson RP. Psychometric properties of the 42-item and 21-item versions of the Depression Anxiety Stress Scales in clinical groups and a community sample. Psychol Assess. 1998. 10.1037/1040-3590.10.2.176

[35] Kroenke K, Spitzer RL, Williams JBW. The PHQ-9. J Gen Intern Med. 2001;16: 606–613. 10.1046/j.1525-1497.2001.016009606.x 11556941 PMC1495268

[36] Spitzer RL, Kroenke K, Williams JBW, Löwe B. A Brief Measure for Assessing Generalized Anxiety Disorder. Arch Intern Med. 2006;166: 1092. 10.1001/archinte.166.10.1092 16717171

[37] Zimet GD, Powell SS, Farley GK, Werkman S, Berkoff KA. Psychometric Characteristics of the Multidimensional Scale of Perceived Social Support. J Pers Assess. 1990;55: 610–617. 10.1080/00223891.1990.9674095 2280326

[38] Zimet G. Multidimensional Scale of Perceived Social Support (MSPSS)—Scale Items and Scoring Information. 2016 [cited 12 Aug 2020]. https://www.researchgate.net/publication/311534896_Multidimensional_Scale_of_Perceived_Social_Support_MSPSS_-_Scale_Items_and_Scoring_Information.

[39] Prins A, Bovin MJ, Smolenski DJ, Marx BP, Kimerling R, Jenkins-Guarnieri MA, et al. The Primary Care PTSD Screen for DSM-5 (PC-PTSD-5): Development and Evaluation Within a Veteran Primary Care Sample. J Gen Intern Med. 2016;31: 1206–1211. 10.1007/s11606-016-3703-5 27170304 PMC5023594

[40] Hair JFj, Black W, Babin B, Anderson R. Multivariate Data Analysis: A Global Perspective (7th ed.). New Jersey: Pearson Education Inc.; 2010.

[41] Czeisler MÉ, Tynan MA, Howard ME, Honeycutt S, Fulmer EB, Kidder DP, et al. Public Attitudes, Behaviors, and Beliefs Related to COVID-19, Stay-at-Home Orders, Nonessential Business Closures, and Public Health Guidance—United States, New York City, and Los Angeles, May 5–12, 2020. MMWR Morb Mortal Wkly Rep. 2020;69: 751–758. 10.15585/mmwr.mm6924e1 32555138 PMC7302477

[42] NYC Health. COVID-19: Data. 2020 [cited 12 Aug 2020]. https://www1.nyc.gov/site/doh/covid/covid-19-data.page.

[43] Chiuve SE, Fung TT, Rimm EB, Hu FB, McCullough ML, Wang M, et al. Alternative Dietary Indices Both Strongly Predict Risk of Chronic Disease. J Nutr. 2012;142: 1009–1018. 10.3945/jn.111.157222 22513989 PMC3738221

[44] Patel A V, Bernstein L, Deka A, Feigelson HS, Campbell PT, Gapstur SM, et al. Leisure Time Spent Sitting in Relation to Total Mortality in a Prospective Cohort of US Adults. Am J Epidemiol. 2010;172: 419–429. 10.1093/aje/kwq155 20650954 PMC3590043

[45] Goldstein RB, Smith SM, Chou SP, Saha TD, Jung J, Zhang H, et al. The epidemiology of DSM-5 posttraumatic stress disorder in the United States: results from the National Epidemiologic Survey on Alcohol and Related Conditions-III. Soc Psychiatry Psychiatr Epidemiol. 2016;51: 1137–1148. 10.1007/s00127-016-1208-5 27106853 PMC4980174

[46] Hasin DS, Grant BF. The National Epidemiologic Survey on Alcohol and Related Conditions (NESARC) Waves 1 and 2: review and summary of findings. Social Psychiatry and Psychiatric Epidemiology. 2015. 10.1007/s00127-015-1088-0 26210739 PMC4618096

[47] Brewin CR, Andrews B, Valentine JD. Meta-analysis of risk factors for posttraumatic stress disorder in trauma-exposed adults. J Consult Clin Psychol. 2000. 10.1037//0022-006x.68.5.748 11068961

[48] Shi S, Qin M, Shen B, Cai Y, Liu T, Yang F, et al. Association of Cardiac Injury with Mortality in Hospitalized Patients with COVID-19 in Wuhan, China. JAMA Cardiol. 2020. 10.1001/jamacardio.2020.0950 32211816 PMC7097841

[49] Zhang SX, Wang Y, Rauch A, Wei F. Unprecedented disruption of lives and work: Health, distress and life satisfaction of working adults in China one month into the COVID-19 outbreak. Psychiatry Res. 2020;288: 112958. 10.1016/j.psychres.2020.112958 32283450 PMC7146665

[50] Shanahan L, Steinhoff A, Bechtiger L, Murray AL, Nivette A, Hepp U, et al. Emotional Distress in Young Adults During the COVID-19 Pandemic: Evidence of Risk and Resilience From a Longitudinal Cohort Study. Psychol Med. 2020. 10.1017/S003329172000241X 32571438 PMC7338432

[51] Castle J, Phillips WL. Grief rituals: Aspects that facilitate adjustment to bereavement. J Loss Trauma. 2003.

[52] Lobb EA, Oldham L, Vojkovic S, Kristjanson LJ, Smith J, Brown JM, et al. Frontline grief: The workplace support needs of community palliative care nurses after the death of a patient. J Hosp Palliat Nurs. 2010.

[53] Bernstein D. Two Decades Ago, The FDA and NIH Mandated the Use of Race to Categorize Subjects and Report Results in Medical and Scientific Research They Oversee. It was a Huge Mistake. Yale J Regul. 2020. Available: https://www.yalejreg.com/nc/two-decades-ago-the-fda-and-nih-mandated-the-use-of-race-to-categorize-subjects-and-report-results-in-medical-and-scientific-research-they-oversee-it-was-a-huge-mistake-by-david-e-bernstein/.

[54] Vinkers CH, van Amelsvoort T, Bisson JI, Branchi I, Cryan JF, Domschke K, et al. Stress resilience during the coronavirus pandemic. Eur Neuropsychopharmacol. 2020;35: 12–16. 10.1016/j.euroneuro.2020.05.003 32446705 PMC7211573

