# Correction: Experiences, impacts and mental health functioning during a COVID-19 outbreak and lockdown: Data from a diverse New York City sample of college students

**DOI:** 10.1371/journal.pone.0337819

**Published:** 2025-11-26

**Authors:** Teresa López-Castro, Laura Brandt, Nishanthi J. Anthonipillai, Adriana Espinosa, Robert Melara

In the Abstract, there are errors in the fourth sentence. The correct sentence is: Findings highlight significant impediments to multiple areas of students’ daily life during this period (i.e., home life, work life, social environment, and emotional and physical health) and a proportion of students endorsing clinically significant levels of depression (36.1%) and generalized anxiety (26.5%).

In the Students’ mental health functioning during the COVID-19 pandemic subsection of the Results, there are errors in the third sentence of the first paragraph. The correct sentence is: Thirty-six percent of students exceeded the cut-off score for the PHQ, indicating severe symptoms of depression in the past 2 weeks, and 26.5% reported severe symptoms of anxiety in the same time period.

In the Psychological functioning during lockdown subsection of the Discussion, there are errors in the first three sentences of the first paragraph. The correct sentences are: Our findings pertaining to the mental health during this prolonged confinement are concerning and support the body of research chronicling the psychological toll of pandemics [1]. Clinically significant levels of stress, anxiety, and depression were reported at rates similar to prior published data on COVID-19-affected populations, which have ranged from 15% to 48% for depression and 6 to 51% for anxiety [2]. The similarity of our study’s rates of depression (36.1%) and anxiety (26.5%) to those of prior COVID-19 work is important because the pandemic-related exposures and impacts reported by our sample appear to differ by an order of magnitude, most notably in infection and mortality exposure.

In the Psychological functioning during lockdown subsection of the Discussion, a sentence was omitted. After the sixth sentence, the following sentence should be included: Nevertheless, that the depression and anxiety prevalence in this “hard hit” sample generally converges with other COVID-19 samples suggests a potential plateauing of the pandemic’s negative emotional impact.

There are errors in Table 4. In the Mean score (SD) row, the values in the DASS-21 Stress column should be 11.84 (9.60), the values in the DASS-21 Anxiety column should be 7.90 (8.23), the values in the DASS-21 Depression column should be 12.41 (10.74), the values in the PHQ-9 column should be 9.42 (6.56), and the values in the GAD-7 column should be 7.03 (5.95.) In the Exceeded cut-off, *N* (%) row, the values in the PHQ-9 column should be 328 (36.1) and the values in the GAD-7 row should be 241 (26.5.) Please see the correct Table 4 here.

There are errors in the S1 Table. In the 1. DASS-21 Stress row, the values in the *M* column should be 11.84 and the values in the *SD* column should be 9.60. In the 2. DASS-21 Anxiety row, the values in the *M* column should be 7.90 and the values in the SD column should be 8.23. In the 3. DASS-21 Depression row, the values in the *M* column should be 12.41 and the values in the SD column should be 10.74. In the 4. PHQ-9 row, the values in the *M* column should be 9.42 and the values in the SD column should be 6.56. In the 5. GAD-7 row, the values in the *M* column should be 7.03 and the values in the SD column should be 5.95. Please see the correct S1 Table here.

**Table 4:** Mental health of the surveyed student sample (N = 909).

	DASS-21 Stress	DASS-21 Anxiety	DASS-21 Depression	PHQ-9	GAD-7	PC-PTSD-5	MPSS Total
**Mean score (SD)**	11.84 (9.60)	7.90 (8.23)	12.41 (10.74)	9.42 (6.56)	7.03 (5.95)	2.73 (1.42)	5.26 (1.32)
**Exceeded cut-off, *N* (%)**	–	–	–	328 (36.1)	241 (26.5)	43 (4.7)	554 (60.9)

DASS-21, Depression, Anxiety, and Stress Scale-21; PHQ-9, Patient Health Questionnaire-9; GAD-7, Generalized Anxiety Disorder-7; PC-PTSD-5, Primary Care-PTSD Screen for *DSM-5*; MPSS, Multidimensional Scale of Perceived Social Support.

## Supporting information

S1 Table Means, standard deviations, and correlations with 95% confidence intervals of mental health measures. (DOCX)
