# Supplementary material for: Experiences, impacts and mental health functioning during a COVID-19 outbreak and lockdown: Data from a diverse New York City sample of college students
Source: PLoS One. 2021 Apr 7;16(4):e0249768. doi: 10.1371/journal.pone.0249768 (PMC8026074; doi:10.1371/journal.pone.0249768)
Supplement: S1 File — (DOCX) [file pone.0249768.s002.docx]

**Supplemental Information: Demographics and College Activities Survey Questions**

What is your year of birth?

What sex were you assigned at birth, such as on an original birth certificate?

- Male (1)
- Female (2)

Do you identify as transgender?

- Yes (1)
- No (2)
- I prefer not to answer (3)

Which term do you use to describe your gender identity?

- Woman (1)
- Man (2)
- Trans woman (3)
- Trans man (4)
- Gender queer (5)
- Another identity (please specify) (6) ________________________________________________

What term best describes your sexual orientation?

- Asexual (1)
- Bisexual (2)
- Gay (3)
- Lesbian (4)
- Pansexual (5)
- Queer (6)
- Questioning (7)
- Same Gender Loving (8)
- Straight/ Heterosexual (9)
- Another identity (Please specify)

What is your year in school?

- 1st year undergraduate (1)
- 2nd year undergraduate (2)
- 3rd year undergraduate (3)
- 4th year undergraduate (4)
- 5th year or more undergraduate (5)
- Graduate or professional (6)
- Not seeking a degree (7)

What is your college enrollment status?

- Full-time (1)
- Part-time (2)
- Other (please specify) (3) ________________________________________________

Have you transferred to this college or university within the *last 12 months*?

- No (1)
- Yes (2)

How do you usually describe yourself? (Mark all that apply)

- White (1)
- Black (2)
- Hispanic or Latinx (3)
- Asian or Pacific Islander (4)
- Native American (5)
- Alaskan Native (6)
- Hawaiian Native (7)
- Biracial (8)
- Multiracial (9)
- Other (please specify) (10) ________________________________________________

Are you an international student?

- Yes (1)
- No (2)

What is your relationship status?

- Not in a relationship (1)
- In a relationship but not living together (2)
- In a relationship and living together (3)

What is your marital status?

- Single (1)
- Married (2)
- Domestic Partnership (3)
- Separated (4)
- Divorced (5)

Where did you live **before the COVID-19 campus closure (March 14th, 2020)**?

- Campus residence hall (1)
- Fraternity or Sorority house (2)
- Rental/ apartment (3)
- Parent/ Guardian's homes (4)

Have you **relocated since the COVID-19 campus closure (March 14th,2020)**?

- Yes (1)
- No (2)

Display This Question:

If Have you relocated since the COVID-19 campus closure (March 14th,2020)? = Yes

Where did you relocate to?

________________________________________________________________

Are you a member of a social (not academic) fraternity or sorority?

- Yes (1)
- No (2)

Within the *last 12 months*, have you participated in organized college athletics at any of the following levels?

|  | Yes (1) | No (2) |
| --- | --- | --- |
| Varsity (1) |  |  |
| Club Sports (2) |  |  |
| Intramurals (3) |  |  |

Has this changed **since the COVID-19 campus closure (March 14th 2020)**?

- Has not changed (1)
- No longer participating (2)
- Participating remotely (4)

In the past 12 months, have you been a member of a student club, or did you participate in clubs or activities?

- Yes (5)
- No (6)

Has this changed since the COVID-19 campus closure (March 14th 2020)?

- Has not changed (1)
- No longer participating (2)
- Participating remotely (3)

Were you employed **before t**he COVID-19  campus closure (March 14th 2020)?

- No (1)
- Yes: Full time on campus (2)
- Yes: Part-time on campus (3)
- Yes: Full time, not on campus (4)
- Yes: Part Time, not on campus (5)

Has your employment status changed due to COVID-19?

- No (1)
- Yes (6)

Display This Question:

If Has your employment status changed due to COVID-19? = Yes

If yes, please indicate how your employment status has changed:

- No longer employed (1)
- Working less hours per week (2)
- Working more hours per week (3)

How many friends would you say you have on campus?

- 0-1 (1)
- 2-4 (2)
- 5 or more (3)
- I'm still mostly friends with my high school or neighborhood friends (4)

How many times were you in contact with your friends in a typical week (outside of classes) **before the COVID-19 campus closure (March 14, 2020)?**Please enter a number below:

________________________________________________________________

Were these contacts:

- Mostly in person (1)
- Mostly virtually (e.g. via phone or social media) (2)
- About the same amount of in person and virtual contacts (3)

How many times were you in contact with your friends in a typical week (outside of classes) after the **COVID-19 campus closure (March 14, 2020)?** Please enter a number below:

________________________________________________________________

Are these contacts:

- Mostly in person (1)
- Mostly virtually (e.g. via phone or social media) (2)
- About the same amount of in person and virtual contacts (3)

How would you describe most of your relationships on campus?

- Friends (1)
- Acquaintances (2)
- Classmates (3)
- Other (please specify) (4) ________________________________________________
