# Supplementary material for: Correction: Experiences, impacts and mental health functioning during a COVID-19 outbreak and lockdown: Data from a diverse New York City sample of college students
Source: PLoS One. 2025 Nov 26;20(11):e0337819. doi: 10.1371/journal.pone.0337819 (PMC12654873; doi:10.1371/journal.pone.0337819)
Supplement: S1 Table — (DOCX) [file pone.0337819.s001.docx]

**S1 Table.** Means, standard deviations, and correlations with 95% confidence intervals of mental health measures [Depression, Anxiety and Stress Scales (DASS), Patient Health Questionnaire (PHQ-9), General Anxiety Disorder (GAD-7), and Primary Care PTSD Screen for DSM-5 (PC-PTSD-5)]

|  | ***M*** | **SD** | **1** | **2** | **3** | **4** | **5** | **6** |
| --- | --- | --- | --- | --- | --- | --- | --- | --- |
| **1. DASS-21 Stress** | 11.84 | 9.60 | 1 |  |  |  |  |  |
| **2. DASS-21 Anxiety** | 7.90 | 8.23 | .80^**^  [.88, .98] | 1 |  |  |  |  |
| **3. DASS-21 Depression** | 12.41 | 10.74 | .81^**^  [.69, .76] | .70**  [.50, .57] | 1 |  |  |  |
| **4. PHQ-9** | 9.42 | 6.56 | .69**  [.47, .54] | .61**  [.35, .42] | .77**  [.60, .67] | 1 |  |  |
| **5. GAD-7** | 7.03 | 5.95 | .75**  [.57, .64] | .71**  [.46, .52] | .70**  [.59, .67] | .77**  [.80, .89] | 1 |  |
| **6. PC-PTSD 5** | 2.73 | 1.42 | .38**  [.92, 1.69] | .45**  [1.11, 1.82] | .33**  [.84, 1.75] | .41**  [1.44, 2.52] | .47**  [1.56, 2.49] | 1 |

**indicates *p* < .01
